# In Vitro Screening of Trehalose Synbiotics and Their Effects on Early-Lactating Females and Offspring Mice

**DOI:** 10.3390/antiox13101223

**Published:** 2024-10-11

**Authors:** Hongmei Peng, Yaya Guo, Jianqiang Zhang, Mengqin Hei, Yuanyuan Li, Wenju Zhang

**Affiliations:** College of Animal Science and Technology, Shihezi University, Shihezi 832000, China; 20222013001@stu.shzu.edu.cn (H.P.); guoyaya@stu.shzu.edu.cn (Y.G.); zhangjianqiang@stu.shzu.edu.cn (J.Z.); heimengqin@stu.shzu.edu.cn (M.H.)

**Keywords:** synbiotics, trehalose, oxidative stress, early-lactating, mice

## Abstract

Activities such as childbirth and breastfeeding can cause severe oxidative stress and inflammatory damage to the mother during early lactation, and can affect animal milk production, and the growth and development of offspring. Trehalose alleviates damage to the body by endowing it with stress resistance. In this study, we used trehalose combined with *Lactobacillus plantarum*, *Bifidobacterium longum*, *Bacillus subtilis*, and *Saccharomyces cerevisiae* to explore whether dietary intervention can alleviate oxidative stress and inflammatory damage in early lactation and to evaluate the growth ability, acid production ability, antioxidant ability, non-specific adhesion ability, antibacterial ability, and other parameters to determine the optimal combinations and proportions. The results showed that the synbiotics composed of 2.5% trehalose and 1 × 10^7^ cfu/g of *Bifidobacterium longum* could regulate the gut microbiota, and promote mammary gland development in dams by reducing progesterone (PROG) content in the blood, increasing prolactin (PRL) and insulin-like growth factor-1 (IGF-1) content, enhancing their antioxidant and immune abilities, and effectively increasing the weight and lactation of early lactating dams. In addition, it can also affect the growth of offspring and the development of the intestinal barrier. These results indicate that trehalose synbiotics have great potential in alleviating oxidative stress and inflammatory damage in early lactation.

## 1. Introduction

In 2019, the Food and Agriculture Organization of the United Nations (FAO) released data indicating that with the growth of the global population and changes in consumption habits, the demand for animal products (including meat and milk) has greatly increased. The mammary gland is an important organ for secreting milk and feeding offspring. Its performance during lactation is closely related to the production of animal products [1]. During lactation, the mammary glands mobilise the body’s nutritional reserves and consume a large amount of energy, accompanied by the production of a large number of reactive oxygen species (ROS) [2]. Combined with the oxidative substances produced during previous childbirth activities, a large amount of reactive oxygen species accumulates in the breasts and body but cannot be cleared promptly, leading to oxidative stress in mothers. Oxidative stress can disrupt the secretion of reproductive hormones, thereby inhibiting breast development, reducing animal milk production, and hindering the production of immunoglobulins and cytokines, leading to postpartum diseases, such as milk deficiency and mastitis [3,4]. It can also cause intestinal hypoplasia, inflammatory bowel disease, and irritable bowel syndrome in offspring [5]. Therefore, determining safe and effective dietary strategies is of great significance to prevent oxidative stress damage and promote lactation, thereby improving the growth and production performance of mammalian mothers and their offspring.

Synbiotics are biological products prepared by mixing prebiotics and probiotics in appropriate proportions [6]. Probiotics can selectively stimulate the growth and proliferation of other beneficial bacteria and can exert their antibacterial effects better under the stimulation of prebiotics. Therefore, the effects of synbiotics are usually greater than those of prebiotics or probiotics alone. As a potential prebiotic, trehalose has good antioxidant, antibacterial, and anti-inflammatory properties [7]. For example, after the oral administration of trehalose, the activity of superoxide dismutase (SOD) in animal plasma increases, the gene expression level of inflammatory cytokines increases, the body’s antioxidant capacity is enhanced, and growth performance is improved [8,9,10]. Most probiotics prevent oxidative stress damage in the body by secreting organic acids and microbial peptides. However, their antioxidant capacity is strain-specific. Guo et al. screened eight strains of *Streptococcus mesenterium* and found that the antioxidant capacity of each strain varied depending on the extracellular polysaccharides secreted [11,12,13]. According to previous reports, *Lactobacillus plantarum* AS1 can assist the body’s antioxidant defence system by eliminating ROS by secreting glutathione peroxidase and rebuilding the gut microbiota, thereby improving the antioxidant performance of mice [14]. Synbiotics combine the functions of prebiotics and probiotics. Tang et al. showed that adding synbiotics is more beneficial for improving the production performance and immune ability of broiler chickens than adding prebiotics or probiotics alone, resulting in healthier offspring [15]. Du et al.’s study showed that the improvement of pig antioxidant capacity and intestinal tract by synbiotics is superior to the addition of probiotics or prebiotics alone [16]. Therefore, screening probiotics with antioxidant capacity and combining them with seaweed polysaccharides to form a synbiotic with antioxidant activity is expected to improve oxidative stress in animals during lactation by increasing antioxidant enzyme activity and rebuilding the gut microbiota. However, current research still lacks an exploration of the role of trehalose synbiotics in mammalian oxidative stress.

Therefore, in this study, trehalose combined with four probiotics was selected as a synbiotic, and a comprehensive evaluation was conducted based on growth ability, antioxidant ability, non-specific adhesion ability, and other parameters to select the most suitable combination and proportion of synbiotics. In the later stages, by evaluating the effects of trehalose synbiotics on the mammary glands and intestines of early lactating dams, new ideas are provided to alleviate maternal oxidative stress during early lactation. In addition, because of the direct correlation between maternal lactation performance and offspring development and health status, we investigated the indirect effects of maternal lactation status on offspring intestinal development, antioxidant capacity, and immune ability after the ingestion of trehalose synthons during lactation.

## 2. Materials and Methods

### 2.1. Materials

Trehalose with an effective content of 98% was purchased from Shanghai Yuanye Bio-Technology Co., Ltd. (Shanghai, China). *Lactobacillus plantarum*, *Bifidobacterium longum*, *Bacillus subtilis*, and *Saccharomyces cerevisiae* were purchased from Henan Saibo Biological Technology Co. Ltd. (Zhengzhou, Henan, China) *E. coli K99* was isolated from the Biological Feed Laboratory of the College of Animal Science and Technology, Shihezi University, China. *Staphylococcus aureus* (ATCC6538) and *Salmonella* (ATCC14028) were purchased from Beijing Microbial Culture Collection Center (Beijing, China). Pepsin and trypsin were purchased from Beijing Biotopped Science and Technology Co., Ltd. (Beijing, China). Sugar-free Mann Rogosa Sharpe (MRS) medium, Yeast extract peptone dextrose (YPD) medium, Lysogeny Broth (LB), and MRS medium were purchased from Qingdao Gaokeyuan Haibo Biotechnology Co., Ltd. (Qingdao, China). Total antioxidant capacity (T-AOC) assay, DPPH radical scavenging ability, and hydroxyl radical scavenging ability kits were provided by Suzhou Gerusi Biotechnology Co., Ltd. (Suzhou, China).

### 2.2. Bacterial Culture Conditions

This study used modified artificial culture media, including modified MRS medium, modified YPD medium, and modified LB medium. Modified MRS medium components included the following: trehalose (20 g/L), peptone (10 g/L), beef extract powder (5 g/L), yeast extract powder (4 g/L), sodium acetate (5 g/L), K_2_HPO_4_ (2 g/L), ammonium citrate tribasic (2 g/L), magnesium sulphate (0.2 g/L), manganese sulphate (0.05 g/L), and tween 80 (1 g/L). Modified YPD medium components included the following: peptone (20 g/L), trehalose (20 g/L), and yeast extract (10 g/L). Modified LB medium components included the following: trehalose (20 g/L), tryptone (10 g/L), yeast extract (5 g/L), and NaCl (10 g/L). The detected probiotics included *Lactobacillus plantarum*, *Bifidobacterium longum*, *Bacillus subtilis*, and *Saccharomyces cerevisiae*. Except for the anaerobic cultivation of *Bifidobacterium longum*, the other three probiotics were grown aerobically at 37 °C.

### 2.3. Characterization and Selection of Probiotics as Potential Synbiotics

#### 2.3.1. Growth Characteristics

Probiotics were cultured in the improved artificial culture medium mentioned above. Cultures were grown for 24 h, and at every 3 h interval, the optical density (OD) was measured using a UV–vis spectrophotometer (Shanghai YOUKE, N5000, Shanghai, China) at 600 nm. A pH meter was used to record the pH value (Shanghai BANGWO, Shanghai, China, Wissenschaftlich-Technische Werkstatten GmbH, Weilheim, Germany, 3110 SET 2). The above modified artificial medium without probiotics was tested as a control.

#### 2.3.2. Antioxidant Activity of Bacterial Strains

We prepared the intact cells and the cell-free extracts. The strain was activated in an improved culture medium, incubated at 37 °C for 24 h, and then centrifuged (10,744 g, 20 min, 4 °C). The collected strains were washed three times with phosphate-buffered saline (PBS) and resuspended in PBS. A portion of the suspension was used to prepare an intact cell (IC) solution (OD_600_ = 0.80 ± 0.02). The remaining suspension was sonicated in an ice bath (4 °C, 30 min) for 3 s, stopped for 10 s, and the output power was 60 W. After centrifugation (10,744 g, 15 min, 4 °C), a cell-free extract (CFE) was obtained and stored at −20 °C for later use.

2,2-Diphenyl-1-picrylhydrazyl (DPPH) radical scavenging activity was assessed as described by Xia et al. [7]. The ICs (or CFEs) and DPPH–ethanol solution (0.2 mmol/L) were mixed in a 1:1 ratio. After standing in the dark for 30 min at room temperature (25 °C), the solution was centrifuged (10,744 g, 5 min, 4 °C). A standard curve for the Trolox standard was plotted with five different concentrations of 5, 10, 15, 20, 25, and 30 μg/mL. The standard curve was calculated as y = 2.8486x + 0.7084, and R^2^ = 0.99991, where x is the Trolox equivalent (μg/mL) and y is the DPPH radical scavenging rate (%). The absorbance was recorded at 517 nm. The DPPH radical-scavenging rate was calculated using Equation (1).

DPPH radical scavenging rate (%) = [1 − (A_1_ − A_2_)/A_3_] × 100]%, (1)

where A_1_ is the absorbance of the experimental group, A_2_ is the absorbance of the control group, and A_3_ is that of the blank group.

Hydroxyl radical (OH^−^) scavenging activity was assessed as described by Smirnoff et al. [8]. The ICs (or CFEs), salicylic acid (9 mmol/L), FeSO_4_ solution and H_2_O_2_ solution were mixed in a 1:1:1:1 ratio and incubated at 37 °C for 20 min. The hydroxyl radical-scavenging ability was determined by measuring the absorbance value of absorbance at 510 nm. The hydroxyl radical-scavenging rate was calculated using the following formula:

Hydroxyl radical scavenging rate (%) = [A_1_ − (A_2_ − A_3_)]/A_1_ × 100%, (2)

where A_1_ is the absorbance of the experimental group, A_2_ is the absorbance of the control group, and A_3_ is that of the blank group.

The total antioxidant capacity (T-AOC) assay was performed following the instructions provided in the Total Antioxidant Capacity Assay Kit. In an acidic environment, ICs (or CFEs) and Fe^3+^-Tripyridine Triazine (Fe^3+^-TPTZ) solutions were mixed and incubated at room temperature (25 °C) for 20 min. A standard curve for the Trolox standard was plotted with four different concentrations (5, 10, 15, and 20 nmol/mL). The standard curve was calculated as y = 0.0972x + 0.0042 and R^2^ = 0.9997, where x is the Trolox equivalent (nmol/mL), and y is the absorbance. Total antioxidant capacity was determined by measuring the absorbance value of absorbance at 590 nm.

#### 2.3.3. Adhesion Ability

The cell surface hydrophobicity assay was performed as described previously [17]. Different probiotics were added to modified artificial media for cultivation. Then, the reaction solution was centrifuged (2650 g, 4 min, and 10 °C). The bacteria were washed three times with PBS and resuspended in the same buffer. The concentration of the bacterial suspension was adjusted to an OD at 600 nm of 0.80 ± 0.02. Then, one millilitre of xylene was added to 3 mL of the bacterial cell suspension in a separate test tube, vortexed for 120 s, homogenised, and allowed to rest for 1 h at room temperature (25 °C). The bacterial cell concentration was monitored by measuring OD at 600 nm. Cell surface hydrophobicity was calculated using the following formula:

The hydrophobicity of cell surfaces (%) = [(A_0_ − A_t_)/A_0_] × 100% (3)

The OD values of OD at 600 nm before and after xylene treatment were assumed to be A_0_ and A_t_, respectively.

Auto-aggregation assay: the preparation of the bacterial suspension was similar to that used for the determination of cell surface hydrophobicity. After leaving the bacterial suspension at room temperature (25 °C) for 5 h, the upper layer was removed, and its OD was measured at 600 nm. Auto-aggregation was calculated using the following equation:

Auto-aggregation ability (%) = (1 − A_t_/A_0_) × 100% (4)

The OD values of OD at 600 nm before and after xylene treatment were assumed to be A_0_ and A_t_, respectively.

#### 2.3.4. Tolerance Ability Assay

Preparation of the bacterial suspension: the probiotic was inoculated with a 4% inoculation amount in an improved culture medium, continuously cultivated for 12 h (37 °C, 180 r/min), and then centrifuged (4 °C, 3000 r/min, 5 min). The supernatant was discarded and the pellet was resuspended in the same buffer. The bacterial suspension was diluted to 10^9^ colony-forming units/mL.

Artificial simulated gastric juice (SGF) was prepared according to the method described by Geng et al. [14]. The pH of the mixture was adjusted to 3.0 with 2 M HCl, followed by the addition of pepsin (0.01 g/mL). Then, the removed samples were filtered through a 0.22 μm microporous filter membrane. For the simulated intestinal fluid (SIF), 3.4 g of KH_2_PO_4_ was added to 250 mL of culture medium. The pH was adjusted to 8.0 with 4 g/L NaOH; trypsin (0.01 g/mL) was added and it was allowed to dissolve evenly before filtering through 0.22 μm filter membranes for sterilisation.

The preparation of the artificial simulated intestinal fluid (SIF) required weighing 3.4 g of KH_2_PO_4_ which was added to 250 mL of the culture medium. After adjusting the pH to 8.0 with 4 g/L NaOH, trypsin (0.01 g/mL) was added, and the solution was allowed to dissolve evenly. Then, it was filtered through 0.22 μm filter membranes. Finally, the bacterial suspension and SGF were mixed and incubated at 37 °C for 3 h. Similarly, the bacterial suspension was added to SIF and incubated at 37° C for 2 h. The number of active bacterial cells in the bacterial suspension was measured using a gradient dilution at 0, 2, and 4 h.

#### 2.3.5. Antibacterial Ability

The pathogen, with a 4% inoculum amount in the above-modified medium, was incubated continuously in the incubator for 12 h (37 °C, 180 r/min) and centrifuged (4 °C, 3000 r/min, 5 min). The supernatant was discarded and the pellet was resuspended in the same buffer. The bacterial suspension was diluted to 10^8^ CFU/mL. LB solid culture mediums were inoculated with 50 μL of pathogens. After the surfaces were dried, the media were drilled into four equal-sized wells in LB solid culture medium and inoculated with probiotics (10^8^ cfu/mL); the culture solution was added to these wells and incubated at 37 °C for 48 h. The diameter of the antibacterial zone was measured to determine antibacterial activity.

### 2.4. Determination of the Optimal Ratio of Bifidobacterium longum and Trehalose

#### 2.4.1. Combination of Different Contents of *Bifidobacterium longum* Synbiotics

Based on ordinary MRS broth culture medium, different proportions of trehalose were added to achieve trehalose contents of 1.25%, 2.5%, and 3.75% in the culture medium. The activation of *Bifidobacterium longum* followed a previously described method. Within the optimal range, the dosage of *Bifidobacterium longum* (effective viable count) was set to three groups: low (1 × 10^7^ CFU/g), medium (1 × 10^8^ CFU/g), and high (1 × 10^9^ CFU/g). The synbiotics were prepared as indicated in Table S1.

#### 2.4.2. Antioxidant Activity of Trehalose Synbiotics

According to a previously described method, slight modifications were made to replace IC (or CFE) with different concentrations of trehalose synbiotic solutions, and the antioxidant capacity was measured, including DPPH radical scavenging ability, hydroxyl radical (OH^−^) scavenging ability, and total antioxidant capacity (T-AOC).

### 2.5. In Vivo Evaluation of Trehalose Synbiotics as Candidate Synthons

#### 2.5.1. Animals, Diet, and Management

The ethical approval and consent to conduct all of the animal experimentation was received from the Ethics Committee of the College of Animal Science and Technology at Shihezi University (No. A2023-220).

Pregnant mice were purchased from the Animal Experiment Centre of Xinjiang Medical University. A total of 12 pregnant mice (ICR), 9 weeks of age, were used for the experiments and their average weight was 45.2 ± 1.5 g. The adaptation period is 5 days. They were divided into a control group (Con group) and a treatment group (TB group), with six mice in each treatment group. The control group was fed a basic diet. The TB group was fed a basal diet before delivery and supplemented with trehalose synbiotics after delivery for 7 consecutive days. The trehalose synbiotics consist of 2.5% trehalose and 1 × 10^7^ CFU/g *Bifidobacterium longum*. The trehalose synbiotics were put into a very small amount of drinking water at 8:00 AM every day, and after it melted, a small amount of basic diet (about 6 g) was used to completely absorb the water. Then, the configured diet was put into the mice cage, and the basal diet was given to the dams after they ate all of the food. Due to the significant impact of life activities such as delivery and lactation, we mainly focused on the early stages of lactation. Early lactation in mice occurs between 0–7 days after delivery [6]. After the delivery of pregnant mice, the artificial control of the number of offspring in each group was eight, provided that each group of pregnant mice produced more than 10 offspring. During the experiment, the animals were allowed free access to water and feed.

#### 2.5.2. Detection Index

The mothers and offspring on the 8th day of lactation, and relevant samples and data, were collected. Production indicators (daily weight gain of the mother and offspring, food intake, and milk production) were measured using the weighing method. Lactation was estimated by the weight of the offspring [18]. After weighing the offspring, the offspring were separated from the mothers for 3 h, and after 3 h, the offspring were weighed again to calculate the metabolizing energy of the offspring. Then the offspring and the dams were placed together for 1 h, and the offspring were weighed again. The test was repeated twice a day. Offspring weight was recorded at 9:00 AM (*W*_1_), 12:00 AM (*W*_2_), 1:00 PM (*W*_3_), 4:00 PM (*W*_4_), and 5:00 PM (*W*_5_).

The milk production (g) = [(*W*_3_ − *W*_2_ + (*W*_1_ − *W*_2_)/3) + (*W*_5_ − *W*_4_ + (*W*_3_ − *W*_4_)/3)]/2 (5)

The dams and offspring were euthanized on the 8th day after delivery. For dams, blood samples were collected by eyeball enucleation method, and they were euthanized by cervical dislocation after blood collection. For offspring, the blood was collected after they were euthanized by decapitation. The blood samples were left to rest for 1 h and centrifuged (4 °C, 3000 r/min, 15 min) to separate the serum. Serum physiological and biochemical indicators (lactation-related hormones in mother mice and intestinal permeability in offspring), serum immune indicators (IgA, IgM, IgG, cytokines), and serum antioxidant indicators (SOD, glutathione peroxidase (GSH-Px), catalase (CAT), and malondialdehyde (MDA)) were detected using an enzyme-linked immunosorbent assay (ELISA). The ELISA kit was purchased from Shanghai Enzyme-Linked Biotechnology Co., Ltd. (Shanghai, China). Tissue sections of the mammary glands and intestines of the dams were analysed using haematoxylin and eosin (H&E), and alcian blue periodic acid-Schiff staining (AB-PAS) methods. The measurement method was based on a study by Virginia et al. [11,12].

#### 2.5.3. Genetic Expression

High-quality RNA was extracted from the mammary gland tissues using RNA extraction kits (Magen Biotech Co., Ltd., Guangzhou, China), and templates were synthesised using the PrimeScript RT reagent kit (Accurate Biology Co., Ltd., Changsha, China). The expression levels of the mammary gland development-related genes Prolactin, Whey acidic protein, and *β-cascin* and antioxidant-related genes *Nqo1*, *Prdx1*, *Nrf2*, and *SOD* were quantified by real-time fluorescence quantitative polymerase chain reaction (RT-PCR) with the specific primers listed in Table S2. The *β-actin* gene was used as the internal reference gene.

### 2.6. Gut Microbiota Analysis

Total DNA was extracted from caecal faeces using a DNA extraction kit (Shanghai Meiji Biotechnology Co., Ltd., Shanghai, China). Then, 1% agarose gel electrophoresis was used to detect the concentration and purity of extracted DNA, and the sample concentration was diluted to 1 ng/μL. The amplification of the bacterial 16S rRNA variable regions V3-V4 was performed using specific primer sequences (338F: ACTCCTACGGGAGGCAGCAG, 806R: GGACTACHVGGGTWTCTAAT). A library was built through the Quant-iT™ PicoGreen^®^ dsDNA Assay Kit, after passing Qubit quantification and PCR detection, and then the Illumina MiSeq platform was used for sequencing. Raw sequences were submitted to the NCBI for the Biotechnology Information Sequence Read Archive (accession number PRJNA1152738).

### 2.7. Statistical Analysis

The data were processed using Excel 2016. A Shapiro–Wilk test and one-way analysis of variance (ANOVA) were performed using SPSS 25.0. The study results were expressed as mean ± standard deviation, with * indicating *p* < 0.05 and ** indicating *p* < 0.01. Principal component analysis (PCA) was performed to analyse bacterial data.

## 3. Results

### 3.1. Evaluation of Potential Probiotic–Trehalose Synbiotics Properties

#### 3.1.1. Growth Characteristics

**Figure 1:**
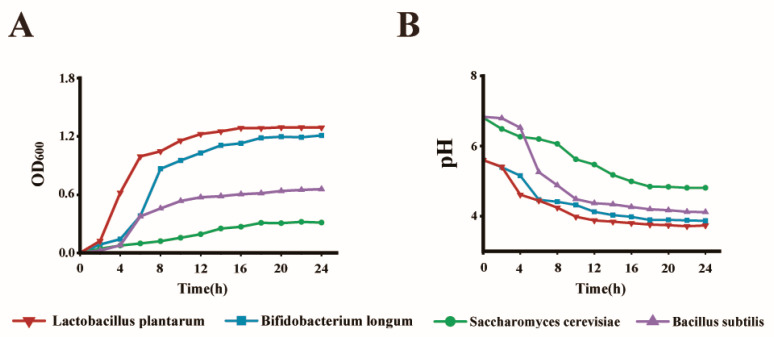
Growth characteristics of different probiotics fermented with prebiotics. (**A**) Growth curve and (**B**) pH profile. The *Lactobacillus plantarum* group, *Bifidobacterium longum* group, *Saccharomyces cerevisiae* group, and *Bacillus subtilis* group represent different combinations of synbiotics synthesized by *Lactobacillus plantarum*, *Bifidobacterium longum*, *Saccharomyces cerevisiae*, and *Bacillus subtilis* with trehalose, respectively.

As shown in Figure 1, all four probiotics utilised trehalose. During the logarithmic period (4–8 h), the growth performance of the *Lactobacillus plantarum* group was excellent, followed by the *Bifidobacterium longum* and *Saccharomyces cerevisiae* groups. *Bacillus subtilis* had the worst utilisation of trehalose. Similarly, the pH change in *Saccharomyces cerevisiae* was the greatest, and acid production was the highest during the logarithmic phase of probiotics. *Bacillus subtilis*, *Bifidobacterium longum*, and *Lactobacillus plantarum* produced less acid than *Saccharomyces cerevisiae*.

#### 3.1.2. Antioxidant Activity

**Figure 2:**
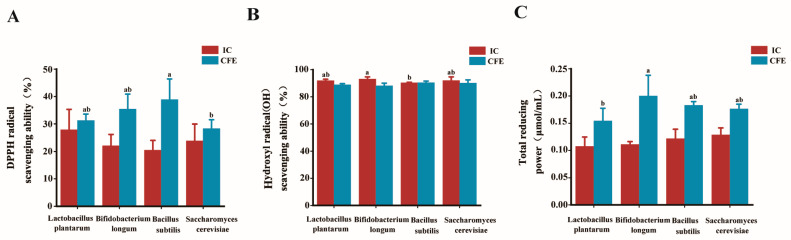
The antioxidant capacity of different strains utilizing trehalose. (**A**) DPPH radical scavenging rate (%); (**B**) hydroxyl radical scavenging ability (%); and (**C**) total restoration capacity (μmol/mL). IC is a complete cell, while CFE is a cell-free extract. Different lowercase letters marked in the figure indicate significant differences (*p* < 0.05).

We evaluated the antioxidant capacities of four combinations of microorganisms. The results of the DPPH radical scavenging assay are shown in Figure 2A. There was no difference in DPPH radical scavenging ability among the synbiotic ICs. However, for CFE, the DPPH free radical scavenging ability of the synthon composed of *Bacillus subtilis* was significantly higher than that of the other synthon combinations, followed by that of the synthon combination composed of *Bifidobacterium longum*. The results of hydroxyl radical (OH^−^) scavenging ability are shown in Figure 2B. Although there was no significant difference in the hydroxyl radical scavenging ability of the CFE of each combination of elements, their scavenging ability was strong (average value ≥ 88%). For IC, the clearance ability of *Bifidobacterium longum* was significantly higher than those of the other groups. As shown in Figure 2C, both IC and CFE exhibited total antioxidant capacity. The total antioxidant capacity of the *Bifidobacterium longum* synbiotic combination was significantly higher than that of the other groups.

#### 3.1.3. Tolerance Assessment

The ARs of the acid resistance of these four groups of probiotics in the presence of trehalose are shown in Figure S1A. At pH 3, 4, and 5, the survival rates of the *Bifidobacterium longum* and *Saccharomyces cerevisiae* groups were significantly higher than those of the other two groups, with the *Bifidobacterium longum* group having the highest survival rate (*p <* 0.05). As pH increased, the survival rate of the strains continued to increase. The bile salt tolerance in the presence of trehalose is shown in Figure S1B. At bile salt concentrations of 0.15%, 0.30%, and 0.60%, the bile salt tolerance of the *Bifidobacterium longum* group and *Bacillus subtilis* group was significantly higher than that of the *Saccharomyces cerevisiae* group (*p* < 0.05). The tolerance to intestinal fluid in the presence of trehalose is shown in Figure S1C. In the simulated intestinal fluid, the concentration of the *Lactobacillus plantarum* group was significantly higher than that in the other three groups at 2 h. At 4 h, the concentrations in *Saccharomyces cerevisiae* group and *Bifidobacterium longum* group were significantly higher than those in the other two groups (*p* < 0.001).

#### 3.1.4. Cell Surface Hydrophobicity Assay and Auto-Aggregation Assay

As shown in Figure S2A, it can be seen that the *Bifidobacterium longum* cultured in the presence of trehalose exhibited the highest hydrophobicity. Similarly, for the automatic aggregation ability (Figure S2B), *Bifidobacterium longum* cultured with trehalose has the strongest automatic aggregation ability.

#### 3.1.5. Antimicrobial Capacity

The ARs of the antimicrobial capacity of these four groups of probiotics in the presence of trehalose are shown in Figure S3. The inhibition results show (Figure S3A) that the probiotic cultures were significantly higher in the *Lactobacillus plantarum* and *Bifidobacterium longum* groups than in the *Saccharomyces cerevisiae* group in terms of the antibacterial effect on *Escherichia coli*. The antibacterial activity of *Saccharomyces cerevisiae* was significantly higher than that of *Bacillus subtilis*. The probiotic cultures were significantly higher in the *Bifidobacterium longum* and *Lactobacillus plantarum* groups than in the other groups in terms of the antibacterial effect on *Staphylococcus aureus*, and the antibacterial ability of *Bifidobacterium longum* was the strongest (Figure S3B). The antibacterial effects of the *Bifidobacterium longum* group, *Lactobacillus plantarum* group, and *Bacillus subtilis* group were significantly higher than those of the *Saccharomyces cerevisiae* group in terms of the antibacterial effect on *Salmonella* (Figure S3C).

#### 3.1.6. Principal Component Analysis (PCA)

The processing was performed as described previously [19]. We evaluated these four combinations of microorganisms to investigate the synergistic effects of trehalose on different bacterial strains. As shown in Tables S3 and S4, three principal components (PC) were extracted that explained 44% of the variance in the cognitive data. PC1 explained 50.975% of the total variation, PC2 explained 26.587%, and PC3 explained 22.438%. Next were *Lactobacillus plantarum*, *Staphylococcus aureus*, and *Bacillus subtilis*.

The correlations between each index level and various syntenic elements are shown in Figure S4. The cumulative contribution rate of these three principal components is 100.000%, representing indicator information. A comprehensive evaluation formula was created using the scores of each principal component and the weights of the variance contributions of each principal component as follows: Y = 0.510Y_1_ + 0.266Y_2_ + 0.224Y_3_. As shown in Table 1, the total score of the *Bifidobacterium longum* group was 1.0747 with the highest score.

#### 3.1.7. Screening of the Optimal Ratio between Trehalose and *Bifidobacterium longum*

We evaluated each combination of synbiotics by measuring the antioxidant capacity of different synbiotic contents (Figure S5). As shown in Figure S5A, the DPPH free radical scavenging ability of the MM group was significantly higher than that of the other groups (*p* < 0.05), and the LM group had the lowest DPPH free radical scavenging ability. For the hydroxyl radical scavenging ability of each group, it can be seen from Figure S5B that the LM group has a significantly higher hydroxyl radical scavenging ability than the other groups (*p* < 0.05), while the LH and HH groups have the lowest DPPH radical scavenging ability. For the total antioxidant capacity of each group, it can be seen from Figure S5C that the total antioxidant capacity of the MH group is significantly lower than that of the other groups (*p* < 0.05). The ranking of each group of antioxidant indicators is presented in Table S5. As shown in Table S5, the ML group ranked first overall; therefore, the combination of 2.5% trehalose and 1 × 10^7^ cfu/g *Bifidobacterium longum* showed the best performance as a synthon.

### 3.2. In Vivo Evaluation of Trehalose Synthons as Candidate Synthons

#### 3.2.1. The Effect of Trehalose Synbiotics on the Postpartum Performance of Mother Mice and Body Weight of Offspring

**Figure 3:**
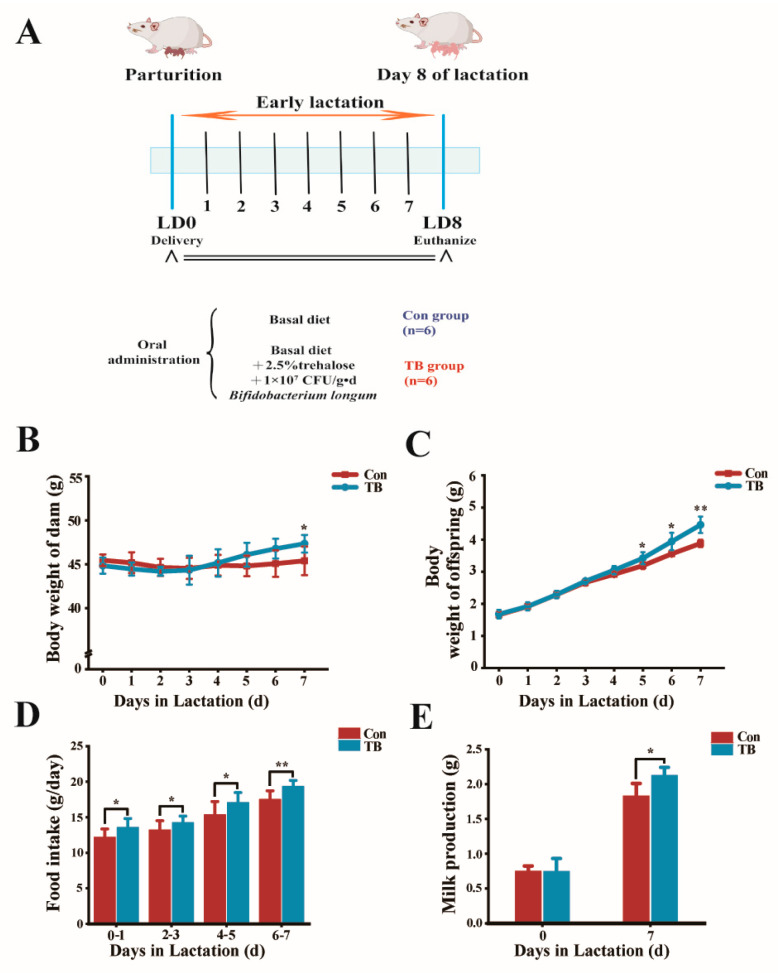
The effect of trehalose synbiotics on postpartum performance of dams and offspring. (**A**) Experimental set-up: In the early stage of lactation (0–7 days after delivery), the dams in the Con group were given a basal diet, and the dams in the TB group were supplemented with trehalose synbiotics on the basis of the basal diet. All rats were euthanized on day 8 after delivery. (**B**) Body weight changes of dams; (**C**) body weight changes of offspring; (**D**) food intake; and (**E**) milk production. * *p* < 0.05; ** *p* < 0.01.

Childbirth and lactation have a great impact on mothers, and it is important to test whether TreS can alleviate the damage caused by reproductive activities. Weight changes in the mother mice are shown in Figure 3A. On the 7th day of early lactation, we found that the weight of the mice in the TB group was significantly higher than that in the control group (*p* < 0.05). Moreover, the weight of the dams showed a trend of first decreasing and then increasing. Changes in feed intake of the dams are shown in Figure 3B. During the lactation period of 0–5 days, the feed intake of the TB group was significantly higher than that of the Con group (*p* < 0.05). On the 6th to 7th day of lactation, the feed intake of the TB group was significantly higher than that of the Con group (*p* < 0.01), showing an overall upward trend. There was no difference in milk production between the two groups of dams on the 0th day of lactation (Figure 3C). With the increase in time, on the 7th day, the milk production of the TB group dams was significantly higher than that of the Con group (Figure 3D). The weights of the offspring in the TB group were higher than those in the Con group on days 5 and 6 (*p* < 0.05). On the 7th day, the body weight of the offspring in the TB group was significantly higher than that in the control group (*p* < 0.01) (Figure 3E).

#### 3.2.2. The Effects of Trehalose Synbiotics on Mammary Gland Development-Related Hormones and Mammary Gland Tissue Morphology

As shown in Table 2, the serum PROG content in the TB group of maternal mice was significantly increased by treatment with trehalose (*p* < 0.05). The PRL content was significantly increased compared to that in the control group (*p* < 0.05). In addition, IGF-1 levels significantly increased (*p* < 0.001).

**Figure 4:**
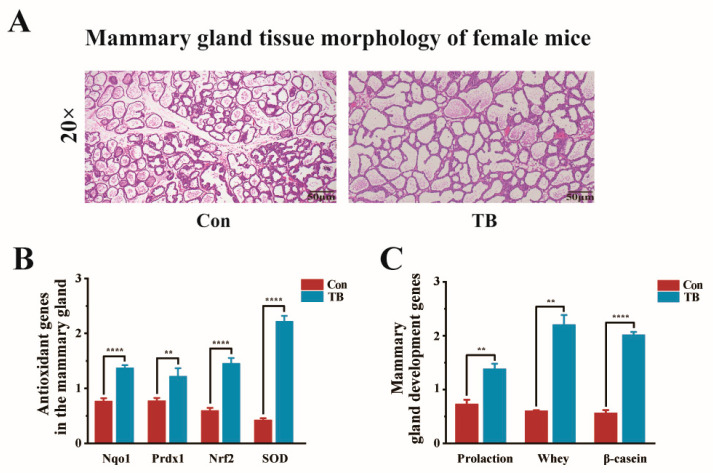
The effects of trehalose synbiotics on the development and antioxidant activity in the maternal mammary gland. (**A**) Representative histologic staining of mammary glands collected from dams. (Original magnification, 20-fold). Scale bars represent 50 μm. (**B**) The expression levels of antioxidant genes (*Nqo1*, *Prdx1*, *Nrf2*, and *SOD*) in the mammary glands of dams. (**C**) The expression levels of development genes (Prolactin, Whey acidic protein, and β-casein) in the mammary glands of dams. ** *p* < 0.01; **** *p* < 0.0001.

However, we also used H&E staining of mammary gland tissue to analyse the histological effects of TreS on the mammary glands of early lactating dams. The distance between the mammary gland acini in the Con group was relatively large, and the structure of the mammary gland acini was irregular (Figure 4A). The mammary gland acini of the TB group were numerous and dense, the acini were neatly arranged and complete, the diameter of the acini was larger, the interacinar space was compact, the mammary lobule was clear, and the duct was thicker.

#### 3.2.3. The Effects of Trehalose Synbiotics on the Immune and Antioxidant Levels of Dams

We tested antioxidant enzyme-related indicators of antioxidant levels in dams. As shown in Table 3, the content of SOD in the serum of mother mice in the TB group significantly increased (*p* < 0.01), the activity of GSH-Px significantly increased (*p* < 0.05), and the concentration of MDA decreased (*p* < 0.05) after the addition of trehalose synbiotics.

We detected the levels of some cytokines (IL-1β, IL-6, IL-10, and TNF-α) and immune proteins (IgM, IgA, and IgG) in the serum of mother mice to investigate whether trehalose synthase can affect the immune level of the dam body. As shown in Table 3, the concentration of pro-inflammatory cytokine IL-1β in the dam serum was significantly reduced and the concentration of anti-inflammatory factor IL-10 significantly increased, due to the use of trehalose synbiotics for treatment (*p* < 0.05). Compared with the Con group, the IgM concentration in the TB group significantly increased with dietary intervention (*p* < 0.001), whereas the IgA concentration significantly increased (*p* < 0.05).

#### 3.2.4. The Effect of Trehalose Synbiotics on the Expression of Antioxidant Genes in the Mammary Gland of Dams

To investigate whether trehalose synbiotics can increase antioxidant capacity and promote mammary gland development, we measured the expression levels of genes related to antioxidants and development in mammary gland tissue. As shown in Figure 4B,C, after treatment with TreS, the mRNA levels of *Nqo1*, *Prdx1*, *Nrf2*, and *SOD* were significantly increased in the mammary gland tissue (*p* < 0.01), whereas the mRNA levels of *PRL*, WAP, and CSN2 were drastically increased by 90% or more.

#### 3.2.5. The Effect of Trehalose Synbiotics on the Gut Microbiota of Dams

**Figure 5:**
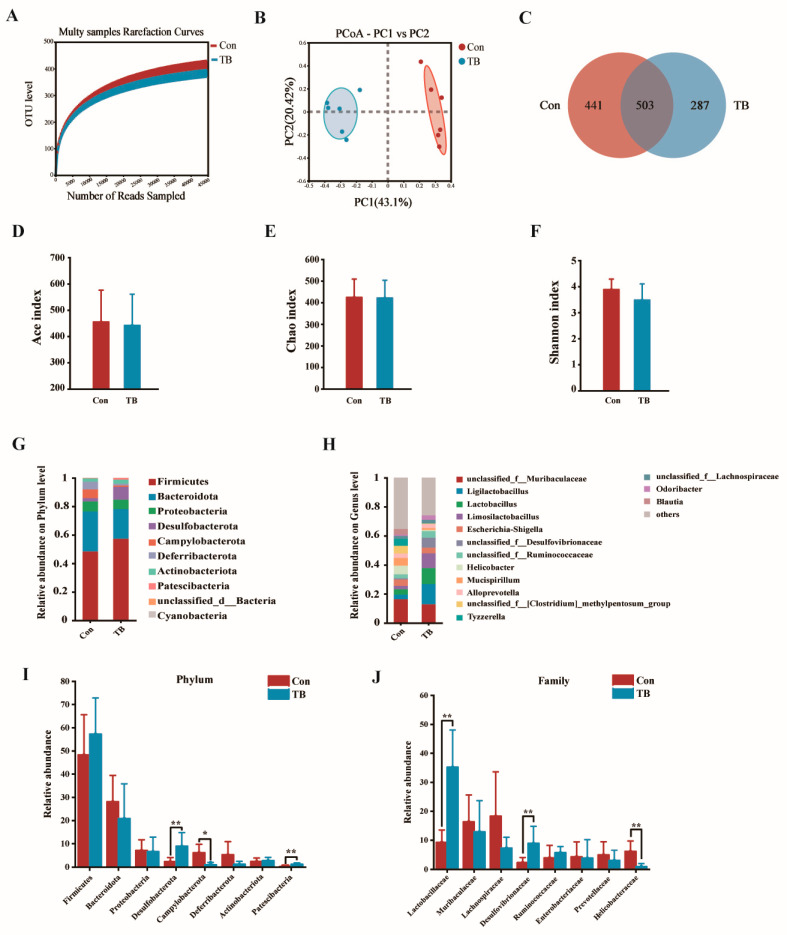
Effects of trehalose synbiotics on gut microbiota in the dam. (**A**) Rarefaction curve. (**B**) PCoA analysis chart. PC1 and PC2 explained variation; the dots represent each sample. (**C**) Venn diagrams showing the OTUs in the two groups: Con, normal control, and TB, trehalose synbiotics. The Con group contained 441 OTUs, the TB group contained 287 OTUs, and there were 503 OTUs in both groups. (**D**) Ace index. (**E**) Shannon index. (**F**) Chao index. (**G**,**H**) Relative abundance of the gut bacterial composition at the level of the phylum and Genus. (**I**,**J**) The relative abundance of gut microbiota at the phylum and species levels. (**I**,**J**) Analysis of differences in dominant microbial communities among groups at the phylum and family levels, with the microbial community ranking among the top eight in relative abundance. * *p* < 0.05; ** *p* < 0.01.

Because TreS is absorbed through the digestive tract and affects the dam body, we speculated that the gut microbiota of mother mice would change with the addition of TreS. We used 16S rRNA gene sequencing to analyse the microbiota in the caecum of mother mice, and the results are shown in Figure 5. The species sparsity curve gradually stabilised with increasing sequencing depth, indicating that the number and depth of sequenced samples met the sequencing requirements (Figure 5A). The differences in the diversity of the intestinal flora among the groups were noted by alpha and beta diversity. Principal coordinate analysis (PCoA) displays the similarity between sample community structures; the greater the distance between samples, the greater the difference in microbial communities between groups [20]. The Con and TB group samples were completely separated and the total explanatory power of PC1 and PC2 for variation was greater than 60% (Figure 5B). The Venn diagram showed 441 operational taxonomic units (OTUs) in the Con group and 287 OTUs in the TB group, with a total of 503 OTUs in both groups (Figure 5C). Although no differences were found in the Ace, Shannon, and Chao indices, the Ace, Shannon, and Chao indices of the TB group were lower than those of the control group (Figure 5D–F). This indicates that trehalose synbiotics can alter the richness and diversity of gut microbiota in mice. To further investigate the effects of trehalose synbiotics on the gut microbiota of dams, we analysed the gut microbiota at the phylum and family levels. At the phylum level, the relative abundances of *Desulfobacterota* and *Patescibacteria* decreased in postpartum mice, whereas the relative abundance of *Campylobacterota* increased. Treatment with trehalose prevented these changes (Figure 5G,I). At the genus level, after treatment with trehalose synbiotics, the abundance of unclassified_f_*Muribaculaceae*, *Escherichia-Shigella*, *Helicobacter*, *unclassified_f_[Clostridium] methylpentosum_group*, and *Tyzzerella* in maternal mice decreased, while the abundance of *Ligilactobacillus*, *Lactobacillus*, and *Limosilactobacillus* increased (Figure 5H). We found a significant increase in *Ligilactobacillus* and *Desulfovibrionaceae*, whereas a significant decrease in *Helicobacteraceae* was observed by analysing the relative abundance of the top eight bacterial communities at the family level (Figure 5J).

**Figure 6:**
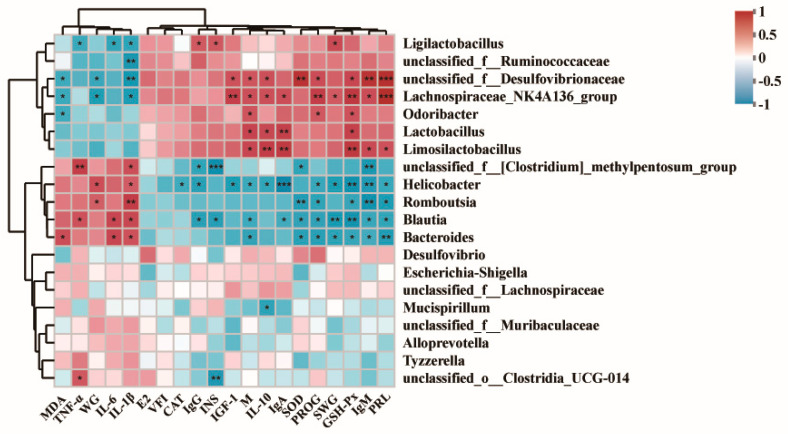
Heatmaps of Spearman correlation analyses. The depth of colour indicates the relative abundance of the top 20 dominant bacterial genera and their correlation with postpartum performance, immunity, and antioxidant activity in dams, with red indicating a positive correlation and blue indicating a negative correlation. MDA: malondialdehyde; TNF-α: tumor necrosis factor-α; WG: body weight changes of dams; IL-6: interleukin-6; IL-1β: interleukin-1β; E2: oestrogen; VFI: food intake; CAT: catalase; IgG: immunoglobulin G; INS: insulin; IGF-1: insulin-like growth factor 1; M: milk production; IL-10: interleukin-10; IgA: immunoglobulin A; SOD: superoxide dismutase; PROG: progesterone; SWG: body weight changes of offspring; GSH-Px: glutathione peroxidase; IgM: immunoglobulin M; PRL: prolactin. * *p* < 0.05; ** *p* < 0.01; *** *p* < 0.001.

The heatmap of the correlation analysis showed that the abundance of *Limosilactobacillus* was significantly (*p* < 0.05) positively correlated with milk production and SOD, GSH-Px, IgA, and IgM levels. The abundance of *Lactobacillus* was significantly (*p* < 0.05) positively correlated with milk production and SOD, GSH-Px, and IgA levels. The abundance of *Limosilactobacillus* was highly significant (*p* < 0.01) and positively correlated with PROG and PRL. The abundance of *Helicobacteraceae* was significantly (*p* < 0.05) positively correlated with milk production, PRL, GSH-Px, IgA, IgM, and IgG, whereas the abundance of *Helicobacteraceae* was significantly (*p* < 0.05) negatively correlated with anti-inflammatory factors such as IL-10. Conversely, this bacteria was significantly (*p* < 0.01) and positively correlated with the pro-inflammatory factors, i.e., IL-1β (Figure 6).

#### 3.2.6. The Effects of Trehalose Synbiotics on the Immune and Antioxidant Levels of Offspring

Numerous studies have shown that the addition of synbiotics can affect the quality of breast milk and, subsequently, the development of offspring. However, it is not yet clear whether the addition of trehalose synbiotics affects the offspring. Therefore, we tested the levels of immune- and antioxidant-related indicators in the serum of offspring. As shown in Table S6, after treatment with trehalose synbiotics, the SOD concentration in the serum of the offspring in the TB group significantly increased (*p* < 0.01), and GSH-Px activity also significantly increased (*p* < 0.05).

We detected the levels of some cytokines (IL-1β, IL-6, IL-10, and TNF-α) and immune proteins (IgM, IgA, and IgG) in the immune capacity of offspring. The results showed that the concentration of IL-10 in the serum of offspring in the TB group was significantly higher (*p* < 0.05). In addition, compared with the young mice in the Con group, the serum concentrations of IgM and IgA in the serum of offspring in the TB group were significantly increased (*p* < 0.01).

#### 3.2.7. The Effect of Trehalose Synbiotics on Intestinal Permeability and Ileal Tissue Morphology in Offspring

We measured indicators related to intestinal permeability to investigate whether adding trehalose synbiotics to the mother could promote intestinal development in the offspring. As shown in Table S7, the concentration of *D*-lactate in the serum of the offspring in the TB group was significantly lower than that in the control group (*p* < 0.05). The concentration of diamine oxidase (DAO) also significantly decreased and the activity of DAO decreased significantly (*p* < 0.001).

**Figure 7:**
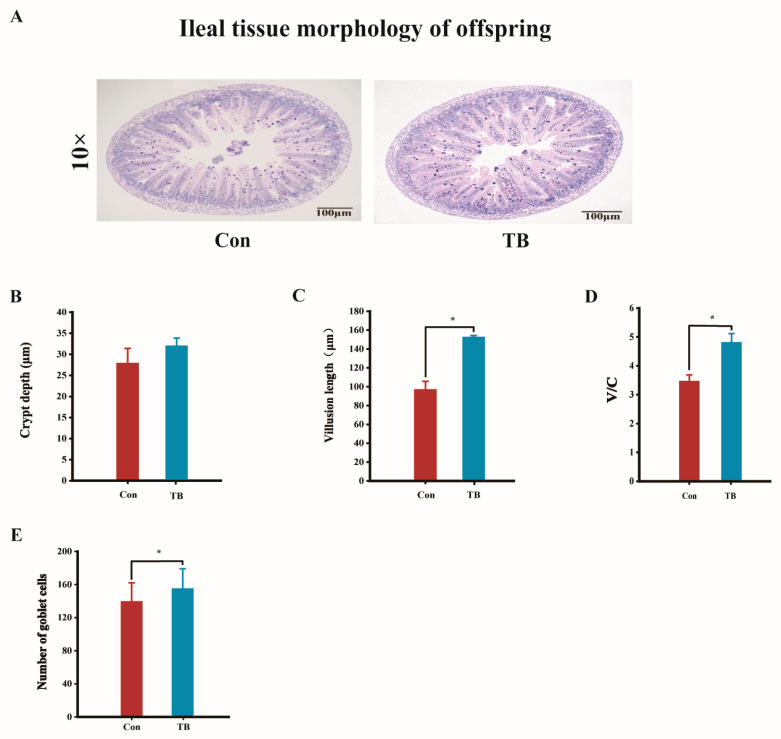
The effects of trehalose synbiotics on the intestinal tract of offspring. (**A**) Representative histologic staining of ileal tissue collected from offspring. (Original magnification, 10-fold). Scale bars represent 100 μm. (**B**) Crypt depth in the ileum of offspring. (**C**) Villus length in the ileum of offspring. (**D**) The villus length-to-crypt depth ratio in the ileum of offspring. (**E**) The number of goblet cells in the ileum of offspring. * *p* < 0.05.

We found that the ileal villus height significantly increased (*p* < 0.05), the crypt depth showed an increasing trend, and the ratio of the ileal villus height to crypt depth significantly increased in the slices of ileal tissue of the offspring in the TB group (*p* < 0.05) (Figure 7A–D). Compared to the control group, the number of goblet cells in the ileum of the TB group was significantly higher (*p* < 0.05) (Figure 7E).

## 4. Discussion

### 4.1. The Most Suitable Combination and Proportion of Trehalose Synbiotics

Trehalose, a potential prebiotic, can help the body resist oxidative damage while being utilised by the gut microbiota [9]. A study found that supplementing cows with trehalose increases SOD activity in the blood and milk [21]. Miura et al. noted that the ingestion of trehalose by calves alters the gut microbiota composition [8]. In a study by Fan et al., trehalose increased the number of lactic acid bacteria in the intestines of broiler chickens and reduced their colonisation by pathogenic bacteria [9]. Compared to ordinary carbon sources, prebiotics cannot be directly utilised by the body, but probiotics can maximise their functions and have unique probiotic effects [22]. Identifying the correct combination of prebiotics is crucial because prebiotics and probiotics are mutually selective. Therefore, we evaluated the best probiotic that matched trehalose from multiple perspectives. A sufficient number of probiotics can work, and probiotics can produce acid to maintain the pH value of the body and kill pathogenic bacteria [23,24]. The results of this study show that different probiotics have different effects on trehalose utilisation. The antioxidant capacity, to a certain extent, represents the protective ability of synbiotics in the body [25]. The results of this study indicate that the combination of *Bifidobacterium longum* and trehalose exerts antioxidant effects. We believe that the entire synbiotic system has antioxidant capacity, but bacterial cells may contain more active substances that scavenge free radicals [22,26]. The hydrophobicity and self-aggregation results indicate that in the presence of trehalose, *Bifidobacterium longum* can better exist in the intestine, which may be related to trehalose being a proliferation factor for *Bifidobacterium* [6,27]. The *Bifidobacterium longum* group had the strongest antibacterial activity, which was similar to the antioxidant results. This indicates a correlation between antibacterial and antioxidant abilities [28]. To select the most suitable combination of synbiotics, we conducted a PCA based on the results of various indicators. We found that the combination of trehalose and *Bifidobacterium longum* exerted maximum antioxidant activity, effectively enhancing its probiotic effect. As previously reported, trehalose can serve as a proliferation factor for *Bifidobacterium longum*, greatly promoting its growth and exerting a protective effect on the body, which is similar to our results [6].

The higher or lower the trehalose and bifidobacterial content, the better the effect [9,21,29]. Therefore, we investigated the optimal ratio of trehalose to bifidobacteria based on their antioxidant properties. The results showed that optimal antioxidant capacity was achieved when the trehalose content was 2.5% of the basal diet and the amount of *Bifidobacterium longum* was 1 × 10^7^ cfu/g·d. Prebiotics are ideal dietary supplements for breeding animals, which can stimulate the growth and metabolism of probiotics. However, due to the different environmental responsiveness and substrate utilization abilities of the strains, there are also differences in their sensitivity to trehalose, which is consistent with our results [30,31]. Therefore, we speculate that at this concentration, *Bifidobacterium longum* has a higher response and utilization ability to trehalose.

### 4.2. The Effect of Trehalose Synbiotics on the Growth Performance and Milk Production of Early Lactating Dams

After childbirth, even while the dam has not yet recovered it needs to feed its offspring, which increases its energy burden. To meet the needs of mammary gland feeding, in addition to consuming energy from foods, the dam needs to consume its own stored energy [32,33]. Synbiotics have been proven to improve animal growth and feed intake. Śliżewska et al. observed that synbiotics have a beneficial effect on the growth of broiler chickens [23]. Ferreres-Serafini et al. noted that synbiotics in piglets can successfully increase their feed intake by increasing the expression of digestive genes [34]. In this study, we observed that the overall weight of the mothers in the pre-lactation stage first increased and then decreased. However, after treatment with trehalose synbiotics, the overall weight loss of the dam decreased, and weight recovery was faster. Moreover, on the 7th day of lactation, the weight of the mothers in the TB group significantly increased. The feed intake of dams in the TB group was higher than that of the control group at the early lactation stage, and the corresponding lactation amount significantly increased on days 6–7. At the same time, we measured the weight of the offspring and found that at 0–4 days, the weights of the two groups of offspring were almost equal, which may be because the mothers in the untreated group consumed more energy to provide nutrition to the offspring. After the 5th day, the growth and development of the offspring in the trehalose synbiotic treatment group was significantly accelerated, and by the 7th day, the weight of the offspring increased significantly. This does not exclude gender influence as a factor. But overall, these findings suggest that trehalose synbiotics can alleviate energy loss caused by childbirth and lactation, increase maternal feed intake and lactation, and promote offspring growth and development.

### 4.3. The Effect of Trehalose Synbiotics on the Development of Mammary Glands in Early Lactating Dams

The mammary gland, an important organ in mammalian reproduction, is an accessory skin gland evolved by the mother to nurture the offspring [4]. It is primarily composed of ducts and acini that form a dendritic branching network [35]. According to previous studies, mammary gland development and lactation are mainly regulated through the hypothalamic–pituitary–mammary axis [10]. Oestrogen (E2) can directly act on mammary gland acinar epithelial cells, stimulate their proliferation, and promote the development of mammary gland acini [36]. During pregnancy, high progesterone (PROG) concentrations promote mammary gland development and milk secretion [37]. After delivery, the PROG concentration decreased in preparation for the initiation of lactation. After childbirth, the secretion of prolactin (PRL) increases because of a significant decrease in the concentrations of oestrogen and progesterone in the blood. As the most critical hormone in the initiation of lactation, its content determines the quality of lactation [38]. Insulin (INS) enhances the nutrient uptake ability of mammary gland cells, thereby promoting their proliferation [4]. Insulin-like growth factor-1 (IGF-1), an important growth factor in mammary acinar epithelial cells, promotes proliferation and inhibits cell apoptosis [39]. Therefore, E2, PROG, PRL, INS, and IGF-1, which are important hormones involved in regulating lactation, may reflect a mother’s lactation ability to some extent. We found that trehalose synbiotics significantly reduced progesterone levels in the maternal serum and significantly increased PRL and IGF-1 levels. In addition, the level of lactation is related to the number and size of the mammary gland acini [40]. We made slices of the dam’s mammary gland to study its development more intuitively. By observing the mammary gland tissue slices of mother mice, we observed that after treatment with trehalose synbiotics, the mammary gland acini of mice were arranged more tightly and had a larger number of acini, the thickness of the mammary gland wall increased, and the mammary gland acini were filled with a large amount of milk. Based on the levels of various hormones in mouse serum, we inferred that trehalose synbiotics could greatly promote the development of mammary gland tissue in maternal mice, promote the proliferation of mammary gland epithelial cells, and improve lactation ability.

### 4.4. The Effect of Trehalose Synbiotics on the Antioxidant and Immune Abilities of Early Lactating Dams

During reproductive activities, such as childbirth and lactation, changes in the maternal environment and enhanced mammary gland metabolism can easily cause the body to produce a large amount of ROS [41]. When there is an imbalance between the production and clearance of ROS in the body, it can lead to the accumulation of ROS in the body, causing oxidative damage to the body’s tissues [1,42]. Trehalose and *Bifidobacterium longum* have been shown to possess good antioxidant capacity [32,43]. SOD converts free superoxide radicals and prevents oxidative damage [44]. GSH-Px converts peroxides into harmless substances via reduction reactions [45]. CAT is an antioxidant enzyme that can degrade hydrogen peroxide and protect the cells from damage. When the body experiences oxidative stress [46]. Therefore, the serum levels of SOD, GSH-Px, CAT, and MDA can reflect the antioxidant levels of the body. In our study, it was found that trehalose synthase significantly increased the levels of SOD and GSH-Px in maternal serum, reducing the concentration of MDA. This indicated that TreS could significantly improve maternal antioxidant levels after delivery. Based on the immune results, we inferred that trehalose synbiotics can enhance the physical fitness of postpartum mice and have significant probiotic potential.

After childbirth, the body is damaged due to childbirth, and the activity of immune cells in the body increases. IL-1β, a key mediator in the inflammatory response, can collaborate with other inflammatory cytokines to promote the occurrence of inflammatory reactions, seriously affecting the proliferation and development of the mammary gland [47]. As an anti-inflammatory factor in the body, IL-10 can reduce the activity of pro-inflammatory factors, promote tissue repair and regeneration, and maintain the immune system [48]. Previous studies have shown that synbiotics can stimulate the body’s immune response by regulating the levels of immune proteins and cytokines to enhance the body’s immune capacity [49]. In this study, we found that after treatment with trehalose synbiotics, the levels of pro-inflammatory cytokines in maternal mouse serum decreased, especially IL-1β. The anti-inflammatory cytokine IL-10 was significantly reduced in the control group, but its levels increased after treatment with trehalose synbiotics. IgM mainly plays a role in the early stages of the immune response as it can neutralise the activity of pathogens and activate the complement system, initiating the body’s immune response [50]. IgA mainly acts on the surface of the mucosa, which can reduce the damage caused by toxins to the body and help offspring establish early immune defences through mammary gland milk [51]. The content of immune proteins in the body increased after treatment with TreS, and the levels of IgM and IgA increased significantly, indicating that TreS can improve the immune ability of early lactating mice.

### 4.5. The Effect of Trehalose Synbiotics on the Expression Levels of Antioxidant Genes and Lactation Genes in the Mammary Gland of Early Lactating Dams

Since lactation is measured by changes in the weight of offspring, this may be influenced by indirect factors such as appetite [34]. To further elucidate the effect of trehalose synbiotics on lactation, we determined the genes related to mammary gland development. The antioxidant capacity of the mammary gland can help cells resist damage caused by free radicals generated by reproductive activities. *Nrf2* is a key transcription factor that maintains the antioxidant balance and signal transduction in the body. It can regulate the expression of downstream antioxidant genes such as *Nqo1*, *Prdx1*, and *SOD* [52]. As a target enzyme of *Nrf2*, *Nqo1* reduces the production of oxidative products in the body and promotes their excretion [53,54]. *Prdx1* is an important member of the peroxiredox protease family that produces ROS and protects cells from oxidative stress damage [55]. *SOD* eliminates superoxide anion free radicals produced by the body and maintains normal cell structure and function [56,57]. In the present study, the expression levels of *Nrf2*, *Nqo1*, *Prdx1*, and *SOD* were significantly upregulated after treatment with alginate synbiotics. *PRL* is a key hormone that initiates lactation, and its expression level is closely related to lactation volume [58]. WAP participates in the encoding of whey proteins and is associated with breast development [59,60]. CSN2, a marker of mammary gland secretory differentiation, regulates milk production [61,62]. In the present study, we found that trehalose synbiotic significantly increased the expression of *PRL*, WAP, and CSN2. This indicates that trehalose synbiotics not only enhance the antioxidant capacity of the mammary gland but also have a positive effect on promoting mammary gland development.

### 4.6. The Effect of Trehalose Synbiotics on the Gut Microbiota of Early Lactating Mother Mice

Recent studies have shown a close relationship between the mammary glands and intestinal microbiota. Similar to the gut–liver and gut–brain axes, there is also a gut–milk axis between the gut microbiota and mammary glands, which mainly functions as a metabolite or migration of the gut microbiota [63]. To confirm whether trehalose synbiotics affected the intestinal environment of dams, we measured the gut microbiota. Our research shows that after treatment with trehalose synbiotics, although there was no significant difference in the alpha diversity index, the overall value was lower than that of the control group. We speculate that this difference may not be significant because of shorter processing times. In addition, the decrease in diversity index values indicates a decrease in microbial richness, which may be due to a reduction in the number and types of harmful bacteria after trehalose synbiotic treatment, promoting the colonisation and growth of beneficial bacteria, consistent with the results of Vu et al. [64]. At the phylum level, we can see that trehalose synthase regulates the gut microbiota of mother mice, with an increase in the ratio of *Firmicutes*/*Bacteroidetes* and a decrease in the abundance of *Campylobacter*. The abundance of *Lactobacillus* increased, whereas that of other pathogenic bacteria in the intestine, such as *Helicobacter* and *Escherichia*-*Shigella*, significantly decreased. This indicates that childbirth can have adverse effects on the intestines of mother mice, and trehalose synbiotics can alleviate this situation by reducing the types and quantities of pathogenic bacteria in the intestines of mother mice, which is also related to the corresponding alpha diversity index results.

### 4.7. Correlation Analysis of Gut Microbiota, Growth Performance, Antioxidant Performance, and Immune Performance in Dams

*Lactobacillus* can affect lactation, immunity, and antioxidant activity in dams. Previous studies have shown that *Limosilactobacillus* (*L. plantarum* Q180 and *L. plantarum* K50) can inhibit mouse mastitis and increase *PRL* expression [65]. Li et al. confirmed that adding *lactobacillus* to sows can reduce the inflammatory response and improve lactation [66]. *Helicobacter* is a pathogenic bacterium that increases the body’s inflammatory response and induces mammary gland cancer [67,68]. In this study, trehalose synbiotic increased the relative abundance of *Limosilactobacillus* and *Lactobacillus* in the gut of dams, while reducing the abundance of *Helicobacteraceae*. Lactic acid bacteria are potential probiotics that affect lactation, immunity, and antioxidant activity in dams. We speculate that trehalose synbiotics improve lactation and immune function by increasing the number of lactobacilli in the gut of dams. This also corresponds with the lactation and immune responses of the mother mice.

### 4.8. The Effects of Trehalose Synbiotics on the Intestinal Development, Antioxidant Capacity, and Immune Function of Offspring

Mammary gland feeding connects mother mice with their newborn offspring, and changes in the dam’s body may affect the offspring [69]. As milk is digested and absorbed through the digestive tract, we studied the antioxidant, immune, and intestinal development levels in the offspring. Although there was no significant change in the MDA concentration in the serum of offspring treated with trehalose synthase, the levels of SOD and GSH-Px significantly increased. The serum test results of the offspring showed a significant increase in the anti-inflammatory factor IL-10 and a significant increase in the levels of IgM and IgA. This is consistent with the results for maternal serum. We believe that this may be related to the immune proteins and cytokines contained in the mother’s body being transmitted to newborn mice through mammary gland milk [70]. These results indicate that the antioxidant and immune levels of the offspring of the trehalose synthon group were greatly improved, indicating that trehalose synthons can affect the immune and antioxidant abilities of offspring by affecting the mother mice. When offspring are born, their intestinal development is not yet mature, and the barrier effect on macromolecular substances, metabolic waste, and toxins is weak [71]. Importantly, intestinal permeability is an important indicator of intestinal integrity and maturity [72]. Under normal circumstances, the concentrations and activities of *D*-LA, DAO, and ET in the blood of offspring are higher than those in the blood of adults. However, after feeding the milk of mother mice treated with trehalose synbiotics, the content or activity of these indicators in the blood of the offspring decreased to a certain extent; in particular, the concentration of *D*-LA and DAO significantly decreased, with a significant decrease in DAO activity. This may be because trehalose synthase improves the milk quality of mother mice and stimulates the rapid development of the intestinal mucosal barrier in the offspring. The ileum is the primary component of the body that absorbs nutrients. Histological sections of the ileum demonstrated the effect of trehalose synthase on intestinal development in the offspring. The results show that treatment with TreS increased the length of intestinal villi, decreased crypt depth, significantly increased the number of goblet cells and mucus levels, and resulted in complete intestinal development. From these results, we can see that the ileal villus height and crypt depth of the offspring of the trehalose synbiotic group increased, and the ratio of villus height to crypt depth increased. The number of goblet cells significantly increased. This indicates that the degree of intestinal development was more complete. We speculate that this is because TreS treatment increased the active substances in maternal milk, promoting the proliferation and development of intestinal cells in the offspring. However, further research is needed to investigate the effects of trehalose on bioactive components in milk, including antioxidants and immunoglobulins. These results indicate that feeding mother mice with trehalose synbiotics can also strengthen the intestinal mucosal barrier function and promote the intestinal development of offspring [73].

## 5. Conclusions

This study identified the best combination of synbiotics (trehalose and *Bifidobacterium longum*). Trehalose not only enhances the activity of *Bifidobacterium longum* but also has good antioxidant activity. It can protect the intestines and mammary glands through the gut–milk axis, enhance the body’s antioxidant capacity, and increase milk production by stimulating mammary epithelial cell proliferation. Thus, trehalose synbiotics may serve as new targets for preventing postpartum oxidative stress. It not only maintains the balance of gut microbiota but also affects the growth and health of offspring through mammary gland milk. Owing to the small number of experimental samples and the short processing time, experimental errors may occur. Further optimisation can be achieved by expanding the sample size.

## Figures and Tables

**Table 1:** Composite score of synbiotics.

Combinations	The Score of Each Principal Component			Total Score Y
	Y1	Y2	Y3	
*Lactobacillus plantarum* group	0.9143	−0.1135	0.2942	0.5019
*Bifidobacterium longum* group	0.8900	0.7088	1.9278	1.0747
*Bacillus subtilis* group	−0.5546	0.8540	0.7200	0.1059
*Saccharomyces cerevisiae* group	−0.1649	0.8561	0.6114	0.2807

The *Lactobacillus plantarum* group, *Bifidobacterium longum* group, *Saccharomyces cerevisiae* group, and *Bacillus subtilis* group represent different combinations of synbiotics synthesized by *Lactobacillus plantarum*, *Bifidobacterium longum*, *Saccharomyces cerevisiae*, and *Bacillus subtilis* with trehalose, respectively.

**Table 2:** Effects on hormones related to mammary gland development in dams.

Item	Experimental Treatments		SME	*p*-Value
	Con	TB		
E2 (pmol/L)	47.02	45.63	0.33	0.065
PROG (ng/mL)	13.51 ^a^	10.43 ^b^	0.51	0.017
PRL (ng/mL)	21.44 ^Bb^	30.91 ^Aa^	1.01	0.002
INS (mU/L)	23.03	24.92	0.44	0.066
IGF-1 (ng/mL)	13.73 ^b^	21.11 ^a^	0.76	0.001

Con, normal control; TB, trehalose synbiotics; oestrogen (E2); progesterone (PROG); prolactin (PRL); insulin (INS); insulin-like growth factor 1 (IGF-1). Different capital letters in the shoulder scale of the same data indicate highly significant differences (*p* < 0.01). Different lower-case letters on the shoulders of the same data indicate significant differences (*p* < 0.05).

**Table 3:** The effects of trehalose synbiotics on the immune and antioxidant levels of dams.

Item	Experimental Treatments		SME	*p*-Value
	Con	TB		
SOD (ng/mL)	45.85 ^Bb^	59.56 ^Aa^	1.79	0.005
GSH-Px (mU/mL)	559.14 ^b^	609.95 ^a^	8.01	0.013
CAT (pg/mL)	49.77	52.64	0.677	0.067
MDA (nmol/mL)	4.51 ^a^	4.06 ^b^	0.096	0.048
IL-1β (pg/mL)	55.93 ^a^	46.80 ^b^	5.56	0.032
IL-6 (pg/mL)	33.16	28.97	1.11	0.097
IL-10 (pg/mL)	27.14 ^b^	30.99 ^a^	0.76	0.035
TNF-α (pg/mL)	241.05	221.44	1.76	0.116
IgM (mg/mL)	13.12 ^Bb^	16.95 ^Aa^	0.38	0.001
IgA (mg/mL)	7.00 ^b^	8.88 ^a^	0.29	0.013
IgG (mg/mL)	27.98	30.61	0.71	0.103

Different capital letters in the shoulder scale of the same data indicate highly significant differences (*p* < 0.01). Different lower-case letters on the shoulders of the same data indicate significant differences (*p* < 0.05).

## Data Availability

The data presented in this study are available on request from the corresponding author.

## Supplementary Materials

The following supporting information can be downloaded at: https://www.mdpi.com/article/10.3390/antiox13101223/s1, Table S1: Combination of different contents of *Bifidobacterium longum* synbiotics; Table S2: Sequence and annealing temperature of primers; Table S3: Characteristic value and the contribution rate of each principal component; Table S4: Extraction results of principal components and component score coefficients; Table S5: Ranking of antioxidant capacity of synbiotics with different contents; Table S6: The effects of trehalose synbiotics on the immune and antioxidant levels of offspring; Table S7: The effect of trehalose synbiotics on intestinal permeability in offspring; Figure S1: The tolerance of probiotics cultured in trehalose medium; Figure S2: The adhesion ability of probiotics cultured in trehalose medium; Figure S3: The antibacterial ability of probiotics cultured in trehalose mediums; Figure S4: The biplot of principal component analysis is based on various indicators; Figure S5: The antioxidant capacity of synbiotics with different contents.
