# Supplementary material for: In Vitro Screening of Trehalose Synbiotics and Their Effects on Early-Lactating Females and Offspring Mice
Source: Antioxidants (Basel). 2024 Oct 11;13(10):1223. doi: 10.3390/antiox13101223 (PMC11505180; doi:10.3390/antiox13101223)
Supplement: Supplementary file 1 [file antioxidants-13-01223-s001.zip › antioxidants-3194951-supplementary.pdf]

Supplementary Table S1

Table S1. Combination of different contents of Bifidobacterium longum synbiotics.

Test groups	Treatments
LL	1.25%Trehalose + Low dose Bifidobacterium longum
LM	1.25%Trehalose + Medium dose Bifidobacterium longum
LH	1.25%Trehalose + High dose of Bifidobacterium longum
ML	2.50%Trehalose + Low dose Bifidobacterium longum
MM	2.50%Trehalose + Medium dose Bifidobacterium longum
MH	2.50%Trehalose + High dose of Bifidobacterium longum
HL	3.75%Trehalose + Low dose Bifidobacterium longum
HM	3.75%Trehalose + Medium dose Bifidobacterium longum
HH	3.75%Trehalose + High dose of Bifidobacterium longum

Table S1 shows the addition of different doses of trehalose and probiotics to regular culture medium.

Supplementary Table S2

Table S2. Sequence and annealing temperature of primers.

Gene	Accession No.	Primer sequence (5′-3′)	Annealing temperature
Nqo1	NM_008706	GTCCATTCCAGCTGACAACC<br>TCCTTTTCCCATCCTCGTGG	60°C
Prdx1	NM_011034.5	CTTCTGTCATCTGGCATGGATTAAC<br>AAGACTCCATAATCCTGAGCAATGG	60°C
Nrf2	NM_010902.5	CAGCATAGAGCAGGACATGGAG<br>GAACAGCGGTAGTATCAGCCAG	60°C
SOD	NM_011434.2	ATGGGGACAATACACAAGGC<br>TCATCTTGTTTCTCGTGGAC	60°C
Prolactin (PRL)	NM_011164.2	CATCAATGACTGCCCCACTTC<br>CCAAACTGAGGATCAGGTTCAAA	60°C
Whey acidic protein (WAP)	NM_011709.5	GCAGCATTTCATGTTGCCA (G)<br>TCGTTCTTGGCCTGCTGGC (C)	60°C
β-casein (CSN2)	NM_001286022.1	ACTCCAGCATCCAGTCACAGC<br>AGGTGAGTCTGAGGAAAAGCC	60°C
β-actin	NM_007393.5	GCTGTCCCTGTATGCCTCT<br>TTGATGTCACGCACGTTT	60°C

Supplementary Table S3

Table S3. Characteristic value and the contribution rate of each principal component.

Principal Components	Eigenvalue	Variance contribution rate（%）	The Cumulative contribution of variance（%）
1	6.627	50.975	50.975
2	3.456	26.587	77.562
3	2.917	22.438	100.000

Supplementary Table S4

Table S4. Extraction results of principal components and component score coefficients.

Variables	Component Matrix			Component score coefficient matrix		
	Principal Components			Principal Components		
	1	2	3	1	2	3
Growth characteristics	0.945	-0.305	-0.121	0.367	0.164	0.071
pH	-0.988	-0.024	0.153	0.384	0.013	0.090
DPPH radical scavenging ability (CFE)	-0.498	-0.457	0.737	0.193	0.246	0.431
DPPH radical scavenging ability (IC)	0.657	-0.315	-0.685	0.255	0.169	0.401
Hydroxyl radical (OH) scavenging ability (CFE)	-0.914	0.402	-0.045	0.355	0.216	0.026
Hydroxyl radical (OH) scavenging ability (IC)	0.883	0.375	0.283	0.343	0.202	0.166
Total reducing power (CFE)	-0.143	0.49	0.86	0.056	0.264	0.504
Total reducing power (IC)	-0.669	0.681	-0.298	0.260	0.366	0.174
Cell surface hydrophobicity assay	0.911	-0.093	0.401	0.354	0.050	0.235
Auto-aggregation assay	0.899	0.118	0.422	0.349	0.063	0.247
Acid resistance	0.51	0.838	0.194	0.198	0.451	0.114
Bile salt tolerance	-0.197	-0.623	0.757	0.077	0.335	0.443
Resistant to intestinal fluid	0.242	0.967	0.081	0.094	0.520	0.047

Supplementary Table S5

Table S5. Ranking of antioxidant capacity of synbiotics with different contents.

Combinations	Rank			Overall ranking
	DPPH radical scavenging ability	Hydroxyl radical (OH) scavenging ability	Total reducing power	
LL	4	6	1	3
LM	9	1	4	5
LH	6	8	4	7
ML	2	3	2	1
MM	1	7	3	3
MH	8	2	5	6
HL	7	4	2	4
HM	3	5	2	2
HH	5	9	4	7

Supplementary Table S6

Table S6. The effects of trehalose synbiotics on the immune and antioxidant levels of offspring.

Item	Experimental treatments		SME	P-value
	Con	TB		
SOD（ng/mL）	42.96[b]	51.80[a]	2.54	0.007
GSH-Px（mU/mL）	495.77[b]	617.03[a]	21.55	0.023
CAT（pg/mL）	49.72	50.14	2.16	0.926
MDA（nmol/mL）	5.52	4.86	0.20	0.134
IL-1β (pg/mL)	28.54	27.31	1.25	0.506
IL-6 (pg/mL)	27.17[b]	30.19[a]	0.50	0.017
IL-10 (pg/mL)	43.06	40.30	1.76	0.455
TNF-α (pg/mL)	255.40[a]	239.03[b]	4.196	0.050
IgM（mg/mL）	14.81	16.08	0.309	0.073
IgA（mg/mL）	6.23[b]	8.46[a]	0.29	0.005
IgG（mg/mL）	22.80[b]	31.91[a]	1.09	0.003

Supplementary Table S7

Table S7. The effect of trehalose synbiotics on intestinal permeability in offspring.

Item	Experimental treatments		SME	P-value
	Con	TB		
D-lactate (μmol/L)	46.99[a]	41.67[b]	1.01	0.031
DAO concentrations(pg/mL)	137.02[a]	108.35[b]	6.08	0.046
DAO activity (U/mL)	17.00[a]	11.96[b]	0.51	0.001
ET(EU/mL)	41.64	37.47	1.39	0.173

Supplementary Figure S1

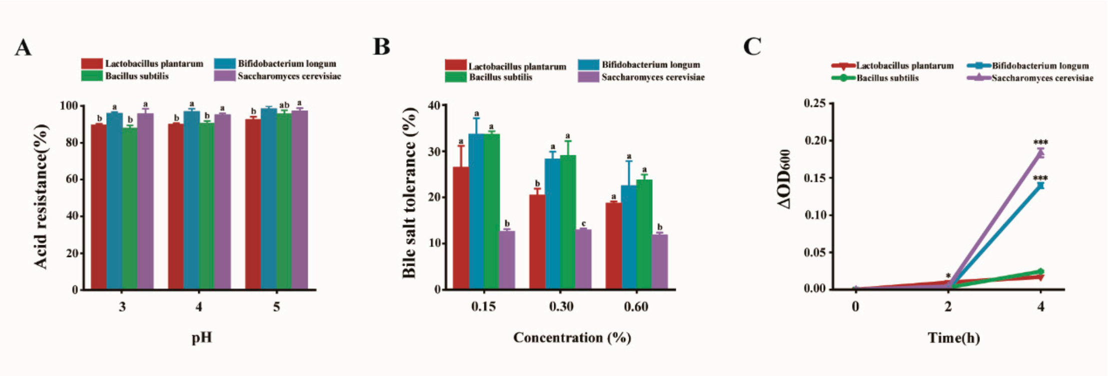

Figure S1. The tolerance of probiotics cultured in trehalose medium. (A) Acid resistance; (B) Bile salt tolerance; (C) Intestinal fluid tolerance. Different lowercase letters marked in the figure indicate significant differences ($p < 0.05$). * $p < 0.05$; ** $p < 0.01$; $p < 0.001$; $p < 0.0001$.

Supplementary Figure S2

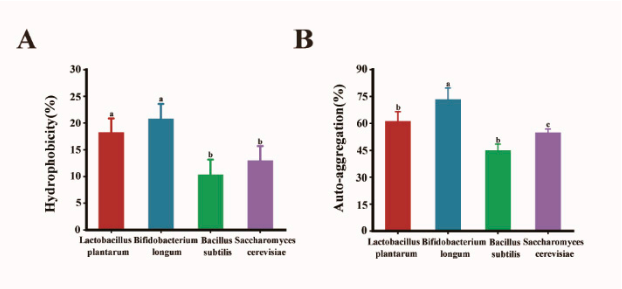

Figure S2. The adhesion ability of probiotics cultured in trehalose medium. (A) Hydrophobicity; (B) Auto-aggregation. Different lowercase letters marked in the figure indicate significant differences ($p < 0.05$).

Supplementary Figure S3

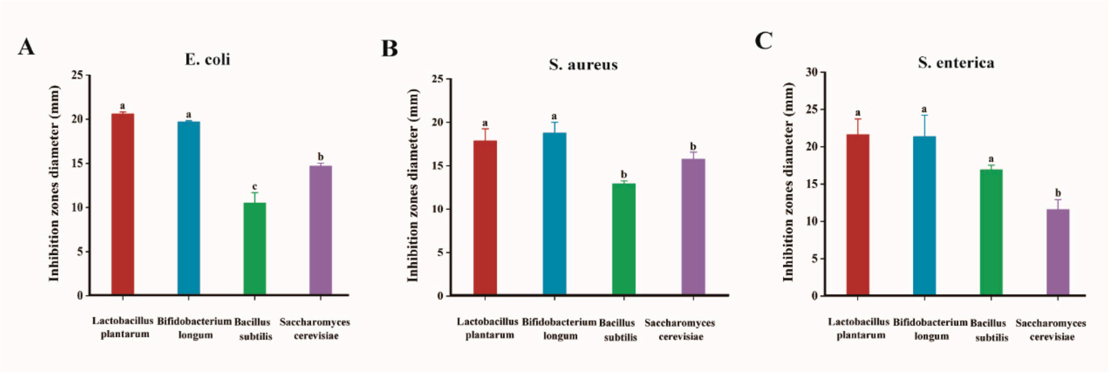

Figure S3. The antibacterial ability of probiotics cultured in trehalose mediums. (A) The antibacterial ability of trehalose synbiotics against Escherichia coli; (B) The antibacterial ability of trehalose synbiotics against Staphylococcus aureus; (C) The antibacterial ability of trehalose synbiotics against Salmonella. The antibacterial ability is expressed in terms of the diameter of zone of inhibition (mm). Different lowercase letters marked in the figure indicate significant differences ($p < 0.05$).

Supplementary Figure S4

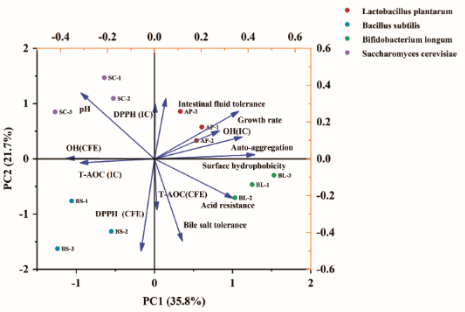

Figure S4. The biplot of principal component analysis is based on various indicators.

Supplementary Figure S5

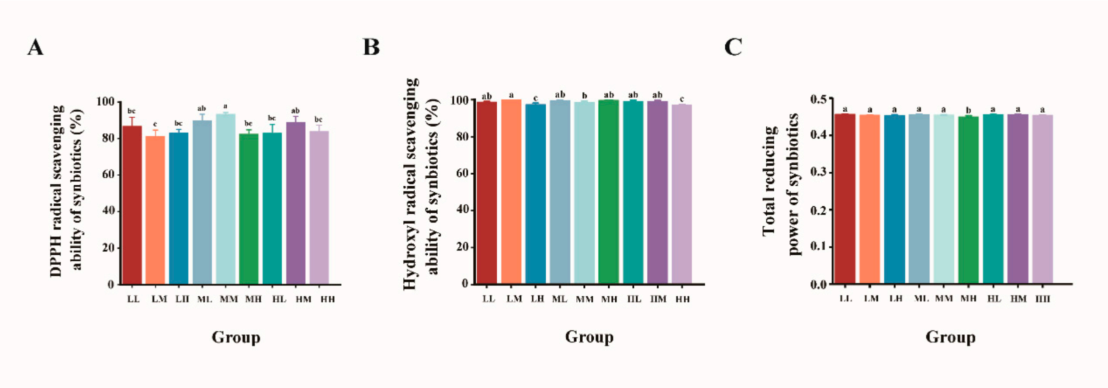

Figure S5. The antioxidant capacity of synbiotics with different contents. (A) DPPH radical scavenging ability of synbiotics with different contents; (B) Hydroxyl radical scavenging ability of synbiotics with different contents; (C) The total antioxidant capacity of synbiotics with different contents. Different lowercase letters marked in the figure indicate significant differences ($p < 0.05$).
